# Ozone-Induced Aryl Hydrocarbon Receptor Activation Controls Lung Inflammation via Interleukin-22 Modulation

**DOI:** 10.3389/fimmu.2020.00144

**Published:** 2020-02-25

**Authors:** Chloé Michaudel, Florent Bataille, Isabelle Maillet, Louis Fauconnier, Cyril Colas, Harry Sokol, Marjolène Straube, Aurélie Couturier-Maillard, Laure Dumoutier, Jacques van Snick, Valérie F. Quesniaux, Dieudonnée Togbe, Bernhard Ryffel

**Affiliations:** ^1^Laboratory of Experimental and Molecular Immunology and Neurogenetics, UMR 7355 CNRS-University of Orleans, Orléans, France; ^2^ArtImmune SAS, Orléans, France; ^3^University of Orléans, CNRS ICOA, UMR7311, F-45067, Orléans, France; ^4^CNRS, CBM, UPR4301, University Orléans, Orléans, France; ^5^Avenir Team Gut Microbiota and Immunity, Equipe de Recherche Labélisée 1157, Institut National de la Santé et de la Recherche Médicale, Paris, France; ^6^Institut de Duve, Université Catholique de Louvain, Brussels, Belgium; ^7^Ludwig Institute for Cancer Research, Université Catholique de Louvain, Brussels, Belgium

**Keywords:** ozone, AhR, ILC3, T cells, inflammation, IL-17, IL-22

## Abstract

Airborne ozone exposure causes severe lung injury and inflammation. The aryl hydrocarbon Receptor (AhR) (1), activated in pollutant-induced inflammation, is critical for cytokine production, especially IL-22 and IL-17A. The role of AhR in ozone-induced lung inflammation is unknown. We report here that chronic ozone exposure activates AhR with increased tryptophan and lipoxin A4 production in mice. AhR^−/−^ mice show increased lung inflammation, airway hyperresponsiveness, and tissue remodeling with an increased recruitment of IL-17A and IL-22-expressing cells in comparison to control mice. IL-17A- and IL-22-neutralizing antibodies attenuate lung inflammation in AhR^−/−^ and control mice. Enhanced lung inflammation and recruitment of ILC3, ILC2, and T cells were observed after T cell-specific AhR depletion using the AhR^CD4cre^-deficient mice. Together, the data demonstrate that ozone exposure activates AhR, which controls lung inflammation, airway hyperresponsiveness, and tissue remodeling via the reduction of IL-22 expression.

**Graphical Abstract:**
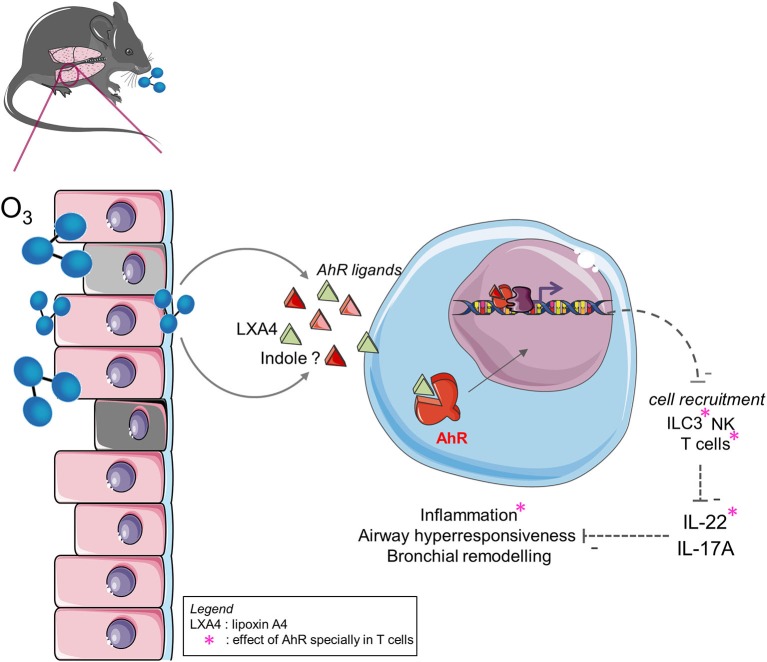
Ozone exposure induces tryptophan metabolites and LXA4 that activates AhR. This activation likely control cell recruitment, T_H_2 and T_H_17/22 response, airway hyperresponsiveness and tissue remodeling with IL-22 dependent manner. AhR activation in lymphocytes influences cells recruitment, IL-22 expression and inflammation.

## Introduction

Ozone is an abundant air pollutant that causes respiratory inflammation. Peaks of ozone correlate with severe respiratory disease, morbidity, mortality (2, 3), and hospital admissions (4–6). An increase of 10 μg/m^3^ of ozone exposure for 1 h daily induces an increase of 0.26% in mortality rate (7). The consequences of ozone exposure are especially deleterious for vulnerable populations with asthma or COPD (8, 9) and exacerbate asthma (10, 11). Ozone causes severe lung tissue damage, with inflammation and emphysema, loss of lung function, and airway hyperresponsiveness in human and mice (12–14).

The aryl hydrocarbon receptor (AhR) (1) is broadly expressed in immune cells and non-hematopoietic cells (e.g., epithelial cells). AhR is implicated in pollutant metabolism and/or degradation in response to polycyclic aromatic hydrocarbons. At steady state, AhR is cytoplasmic. In the absence of ligands, AhR resides in the cytoplasm under the control of a chaperone protein complex. Upon ligand binding, the AhR complex translocates into the nucleus, the chaperones are released, and AhR heterodimerizes with AhR Nuclear Translocator (ARNT) (15). This complex binds to Dioxin Response Elements (14) on the DNA and induces gene transcription, including P450 cytochrome and cytokines (e.g., IL-17A, IL-22). Non-canonical AhR signaling pathways have also been reported, either at the genomic level, through association with other transcription factors (e.g., NF-κB), or at the non-genomic level (e.g., through the release of the Src kinase) (16, 17). AhR is known to mediate several aspects of immune response and homeostatic maintenance, and its action is dependent on the context. For example, AhR can interact with NF-κB and STAT1 to inhibit IL-6 production after LPS treatment (18). AhR activation affects different pathways, such as T cell differentiation and antioxidant response. Furthermore, AhR induces ILC3 maintenance in the gut via Cyp1a1 (19) and triggers T_H_17 cells differentiation, promoting IL-17 and IL-22 production by inhibiting IFN-γ (20). The AhR activity is dependent on context, but also ligands. AhR ligands can be of endogenous (e.g., tryptophan metabolism derivatives from the microbiota) or of exogenous origin (e.g., Benzo[a]pyren, environmental pollutants, and dietary-derived compounds) (21). The response of AhR activation depends on the nature of the ligand. For example, AhR in PBMCs stimulated by TCDD (2,3,7,8-tetrachlorodibenzo-p-dioxine) induces IL-22 and IL-17A production, while stimulation by prostaglandin E2 (PGE2) has the opposite effect (22). AhR's effect on T cell polarization also depends on the nature of the ligand, as it can either promote T_H_17 or Treg cell differentiation (23). Taken together, the literature reported that AhR could have a principally protective effect, for example during major depressive disorder, multiple sclerosis, rheumatoid arthritis (24), and intestinal disease (16).

IL-17A has a pro-inflammatory role in several models of inflammation, such as asthma or colitis (25, 26). IL-17A could be produced by T cells, iNKT, NK, γδ T cells, and ILC3 and could modulate the production of other pro-inflammatory cytokines (e.g., IL-6 and IL-8), chemokines, and molecules involved in tissue remodeling, such as MMPs (27). During acute ozone exposure, IL-17A is produced by γδ T cells and iNKT (28, 29) and induces neutrophil recruitment and airway hyperresponsiveness. Upon chronic ozone exposure, IL-17A also induces inflammation, neutrophil recruitment, and airway hyperresponsiveness (30, 31). Furthermore, IL-17A induces M2 macrophage polarization and promotes apoptotic cell clearance (32). Similarly to IL-17A, IL-22 is produced by αβ T cells (T_H_17, T_H_22), γδ T cells, NK, and ILC3, after stimulation with IL-1β, TGF-β, or IL-23 and the transcription factor RORγt (23, 33, 34). IL-22 is a pro- or anti-inflammatory cytokine, depending on the inflammatory context. IL-22 plays a protective role when produced during epithelial or tissue damage, while IL-17A/IL-22 collaboration promotes IL-22 pro-inflammatory activity (35, 36).

In the current study, we identified an AhR ligand, the lipoxin A4, a tryptophan metabolite, which is produced after chronic exposure to ozone and induces AhR activation in the lung. AhR-deficient mice showed an increased lung inflammation and an increased cytokine production, including IL-17A and IL-22. Consistent with previous observations, we hypothesized that the protective effect of AhR is linked to the indirect repression of IL-17A and IL-22 by the reduction of cell recruitment of NK, T cells, and ILC3. Using T cell-specific AhR-deficient mice (CD4^cre^ AhR^f/f^), we showed that AhR present in T cells induces the indirect repression of IL-22 and IL-17A by mediation of the recruitment of T cells of IL-22^+^ or IL-17^+^ and ILC3 of IL-22^+^ or IL-17^+^. Using IL-22^−/−^, IL-22Rα^−/−^, and IL-22xIL-17Rα^−/−^ mice, we demonstrate the pro-inflammatory role of IL-22 during chronic ozone-induced inflammation (Graphical Abstract). IL-22 blockade in AhR^−/−^ mice restores WT mouse phenotype. Altogether, these findings suggest that AhR activation during ozone exposure may be beneficial for the host, and thus, AhR agonists or IL-22 blockade may represent potential therapeutic strategies in ozone-induced lung inflammation.

## Methods

### Mice and Reagents

AhR^−/−^ (from Frank Gonzalez) (37), AhR^flox/flox^ (from Christopher Bradford), AhR^CD4cre^, IL-22^−/−^ (38), and IL-22 × IL-17Rα^−/−^ (38) mice and littermate controls of 7–9 weeks of age were used for the study. IL22Ra1^tm1a(EUCOMM)Wtsi^ mice were obtained from the International Mouse Phenotyping Consortium via EMMA network. Generation of mice carrying the Il22Ra1^tm1c(KOMP)Wtsi^ and Il22Ra1^tm1d(KOMP)Wtsi^ alleles was achieved by breeding to StellaCre mice expressing Cre (Dppa3^tm1(cre)Peli^) (39) and/or Flp (ROSA26Fki) recombinases. Colonies were maintained on a C57BL/6 genetic background.

All gene-deficient mice and WT (40) controls (C57BL/6J background) were bred and housed in our specific pathogen-free animal facility at Transgenose Institute (TAMM-CNRS, UPS 44 under agreement D-45-234-6, 2014, Orleans, France). In all experiments, 5–6 female mice per group were used, were maintained in a temperature controlled (23°C) facility with a strict 12 h light/dark cycles, and were given free access to food and water. Animal experiments were performed according to the French Institutional Committee under agreement CLE CCO 2015-1088.

Anti-IL-22 antibody (AM22.1, mouse anti-mouse, 14 μg/mouse, from Laure Dumoutier) and anti-IL-17A antibody (MM17AF3, mouse anti-mouse, 20 μg/mouse, from Jacques van Snick) were administered intranasally, once a week for 6 weeks.

### Ozone Induced Airway Inflammation

Mice were exposed to ozone in a plexiglass chamber (*EMB 104, EMMS*®) at 1.5 ppm for 2 h, two times a week for 6 weeks. Ozone was created by an ozonisator (*Ozonisator Ozoniser S 500 mg, Sander*®) and a level of 1.5 ppm was controlled by a sensor (*ATI 2-wire transmitter, Analytical Technology*®). Mice were euthanized by progressive CO_2_ inhalation, 24 h after last ozone exposure and BAL was collected. After a cardiac perfusion with ISOTON II (*acid-free balanced electrolyte solution Beckman Coulter, Krefeld, Germany*), lung was collected and sampled for analyses.

### Broncho Alveolar Lavage (11)

After ozone exposure, BAL was performed by four lavages of lung with 500 μL of saline solution (NaCl 0.9%) each time, via a cannula introduced into mice trachea. BAL fluids were centrifuged at 300 g for 10 min at 4°C, the supernatants were stored at −20°C for ELISA analysis, and cell pellets were recovered to prepare cytospin (*Thermo* S*cientific, Waltham, USA*) on glass slides, followed by a staining with Diff-Quik solution (*Merz & Dade A.G., Dudingen, Switzerland*). Differential cell counts were performed with at least 400 cells.

### DNA Measurement

DNA release was measured to evaluate the tissue damage in the BAL supernatant using the Quant-iT™ PicoGreen™ dsDNA assay kit (*ThermoFisher*), according to the manufacturer's instructions.

### Measurement of Cytokines, Collagen, and Total Proteins

IL-22, IL-17A, MPO, and AREG in BALF were determined by ELISA (*R & D systems, Abingdon, UK*), while IL-4, IL-5 in lung, and TGF-β1 from BAL were measured by Luminex immunoassay using MagPix reader (Bio-Rad) according to the manufacturer's instructions. Data were analyzed with Bio-Plex Manager software (Bio-Rad). Total protein levels in BALF were analyzed with the Bio-Rad DC Protein Assay. Collagen was measured with Soluble Collagen Assay Sircol™ (*biocolor*), according to the manufacturer's instructions on BAL and lung supernatants. For the protein measurement on the lung supernatant, the same part of the lung was harvested and gridded in 1 mL of PBS with proteases inhibitor cocktail (Roche). After centrifugation, supernatant was collected and frozen.

### AhR Ligand Determination

Lung supernatants were mixed with DPBS up to a total volume of 3 mL. SPE (solid-phase extraction) was then performed on Supelclean LC-8 SPE cartridges (*Supelco*) conditioned with methanol and water. The sample was loaded then washed with 3 mL of ultrapure water and dried for 15 min under vacuum (10 mmHg) before being eluted with 5 mL of an 80:20 dichloromethane:isopropyl alcohol mixture. The exudate was evaporated to dryness under a nitrogen flow and reconstituted in 200 μL of a 50:50 acetonitrile:water mixture.

LC-HRMS analyses were performed on a maXis Q-TOF mass spectrometer (*Bruker, Bremen, Germany*) coupled to an Ultimate 3000 RSLC system (*Dionex, Germering, Germany*). The column was a Kinetex C18 (150 × 2.1 mm) with a particle size of 1.7 μm (Phenomenex, Le Pecq, France), equipped with a C18 SecurityGuard Ultra (2.1 mm) guard filter (*Phenomenex*, Le Pecq, France). The mobile phase consisted of a gradient of water with 0.1% of formic acid (solvent A) and acetonitrile with 0.08% of formic acid (solvent B) as follows: 0–0.3 min 3% B, 0.3–10 min 3–90% B, 10–10.5 min 90% B, and finally, 10.5–10.6 min 90–3% B, maintained for 1.5 min before each new injection. The column was thermostated at 40°C, and the flow rate was 500 μL/min. The injection volume was 2.5 μL. The mass spectra were acquired with an ESI (electrospray Ionization) source in positive mode in the range of 50–1,650 *m/z* at a frequency of 1 Hz. The capillary voltage was set at 4.5 kV, the pressure of nebulizing gas was 2.0 bar, and the flow rate of drying gas was 9 L/min, heated at 200°C.

### Histology

The left lung tissue was fixed in 4% buffered formaldehyde and paraffin embedded under standard conditions. Tissue sections (3 μm) were stained with Periodic acid Schiff (41). Histological changes were determined by a semi-quantitative severity score (0–3) for inflammatory cell infiltration (Table 1). The slides (one mouse per slide) were blindly examined by two independent investigators with a Nikon microscope (Nikon eclipse 80i, United States). All mice were scored (1 big and 3 small bronchi per mice).

**Table 1:** Lung inflammation histological scoring.

**Score**	**Cell infiltration**
**0**	No cell infiltration
**1**	Moderate infiltration around vessels
**2**	Moderate infiltration around vessels and bronchi
**3**	High infiltration around vessels and bronchi

### FACS Analysis

Lungs were collected from mice, minced, digested with DNase (Sigma, 1 mg/mL) and Liberase (Roche, 5 mg/mL) during 1 h at 37°C, and red bloods cells were lysed with Lysing buffer (*BD Pharm Lyse*^*TM*^, *BD Pharmingen*). Stimulation was performed with PMA (50 ng/ml, *Sigma*) and ionomycin (750 ng/ml, *Sigma*), for 2 h and 30 min. Cells were incubated with the antibodies for 25 min at 4°C in FACS buffer (PBS, 2% FCS, 2 mM EDTA). For intracellular staining, BD Cytofix/Cytoperm™ was used according to the manufacturer's instructions. Cells were washed with FACS buffer and fixed by lysing buffer 1 × (BD Pharmingen). The antibodies used were against mouse molecules and described in the Table S1. The gating strategy is presented in Supplemental Figure 1 and was conducted as follows: epithelial cells (CD45^−^EpCam^+^), NK cells (CD45^+^NKp46^+^NK1.1^+^), interstitial macrophages (CD45^+^F4/80^+^CD11c^−^), alveolar macrophages (CD45^+^F4/80^+^CD11c^+^), neutrophils (CD45+GR1+), CD4+ T cells (CD45^+^CD3ε^+^CD4^+^), γδ T cells (CD45^+^CD3ε^+^TCRγδ^+^), ILC2 (CD45^+^lin^−^ICOS^+^ST2^+^), and ILC3 (CD45^+^lin^−^RORγt^+^CD127^+^). The lineage staining used was composed of CD11b, CD3ε, CD45R/B220, Siglec-F, and FcεRIα. Data were acquired (~2 million cells) with a flow cytometer (*BD FacsCanto II*) and analyzed with FlowJo software (*TreeStar, Mountain View, CA*).

### *Ex vivo* Stimulation of Lung Cells

Lung cells were collected from mice as described above. Stimulation was performed on 10^6^ cells with PMA (50 ng/ml, *Sigma*) and ionomycin (750 ng/ml, *Sigma*) for 24 h. After stimulation, supernatant was harvested and frozen at −20°C.

### Immunofluorescence

Lungs were treated with paraformaldehyde (42) perfusion, and tissues were fixed for 3 days in PFA 4% and transferred in sucrose 20% for 2 weeks. Half of right lung was included in tissue-tek® O.C.T (*4583, tissue-tek*®) and 10 μm frozen tissue were sectioned by using a cryomicrotome (*Leica*) at −20°C. For immunofluorescence staining, slides were incubated 30 min in citrate buffer at 80°C. Slides were washed with PBS 1× after each step, followed by 45-min incubation in saturation medium (TBS 1× , 1% BSA, 10% SVF, 0.3% triton X100). The primary antibody, an anti-AhR (10 μg/mL, ab2769, Abcam), was incubated overnight at 4°C. After washing, slides were treated with pontamin (0.05%, Chicago sky Blue 6B, Sigma) for 15 min. After washing (TBS 1×), slides were incubated with a secondary antibody, a goat-anti-mouse (4 μg/mL, ab150113, *Abcam*) during 1 h at room temperature. After washing, slides were incubated with DAPI (1/5,000 dilution) for 10 min, washed, and mounted in mowiol® (*Sigma*). Lung sections were observed under a fluorescence microscope Leica (*Leica*®, CTR6000) at 400× magnification for microphotography.

### Lung Function Determined by Invasive Plethysmography

Airway hyperresponsiveness was measured by using increasing concentrations of methacholine (25–200 mg/mL) using the FinePointe system (Buxco, DSI) as previously described (43).

### AhR/Luciferase Reporter Assay

The H1L1.1c2 cell line was used as described before (16). Control (10% NaCl), supernatant of BAL and lung were used and incubated with the H1L1.1c2 cell line. AhR activity was calculated by subtracting the luminescence of the control (10% NaCl) from the luminescence obtained with the samples multiplied by the cytotoxicity value.

### CD4+ T Cells Isolation

CD4+ T cell were isolated from the spleens of WT, AhR^CD4cre^, and AhR^-/-^ mice with Dynabeads™ Untouched™ Mouse CD4 Cells Kit (*ThermoFisher*). 6–7 × 10^6^ CD4+ T cells were extracted from each spleen, and RNA was extracted as described below.

### RNA Extraction, Reverse Transcription and Quantitative Real-Time PCR

Total RNA was extracted using TRIzol reagent (*Sigma*) followed by a phenol/chloroform extraction. Total RNA (1 μg) was reverse-transcribed using GoScript™ Reverse Transcription System (*Promega*). The mRNA levels for the genes of interest were examined by quantitative RT-PCR using the GoTaq® qPCR Master Mix according to the manufacturer's protocol. The primer used were obtained from *Qiagen*: AhR (QT00174251), Cyp1a1 (QT00105756), and AhRR (QT00161693). Relative levels of mRNA expression were normalized to HPRT1 (QT00166768) mRNA levels using a comparative method (2^−ΔΔCt^). Non-reverse-transcribed RNA samples and water were included as negative controls.

### Statistical Analysis

Data were analyzed using Prism version 6 (*Graphpad Software, San Diego, USA*). The non-parametric Mann Whitney test or parametric one-way ANOVA tests with multiple Bonferroni's comparison tests were performed. Values are expressed as mean ± SEM. Statistical significance was defined at a *p*-value ^****^ < 0.0001, ^***^ < 0.001, ^**^ < 0.01, and ^*^ < 0.05. Statistical differences between air and ozone mice in the same genotypes were represented by a star (or ns) just on the top of column of ozone mice, other comparisons are represented with bar between the groups compared.

## Results

### Chronic Ozone Exposure Induces Tryptophan Metabolites Release and AhR Activation

**Figure 1:**
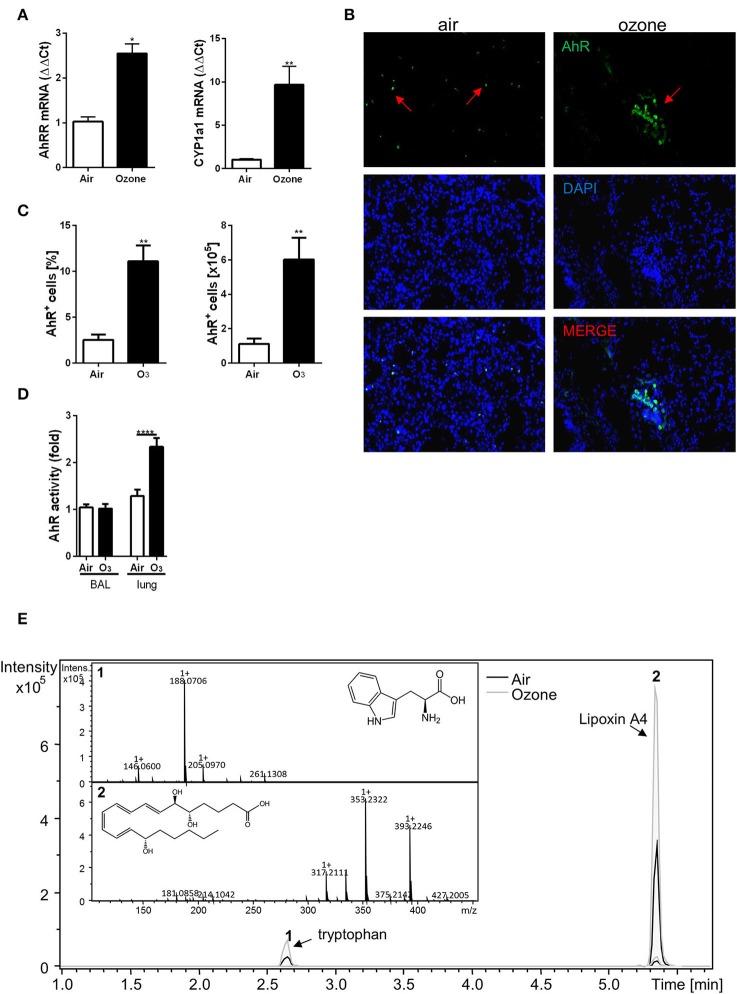
Chronic ozone exposure induces AhR activation. Quantification of AhRR and Cyp1a1 involved in the AhR pathway activation **(A)**. AhR detection in lung, by immunofluorescence staining (magnification 400×; arrows represent AhR^+^ cells) **(B)** and FACS **(C)**. AhR activity in the BAL and the lung after 6 weeks ozone exposure **(D)**. Relative abundance of tryptophan and Lipoxin A4 in lung supernatant analyzed by LC-MS **(E)**. The data are representative of one from two independent experiments with *n* = 5–6 mice per group. Values are expressed as mean ± SEM. Statistical significance was defined at a *p*-value **** < 0.0001, *** < 0.001, ** < 0.01, and * < 0.05.

First, we asked whether AhR is activated upon chronic respiratory ozone exposure. Thus, we investigated the expression of associated AhR genes after chronic ozone exposure (twice weekly to 1.5 ppm for 2 h for 6 weeks). We observed an increased mRNA expression of the AhR repressor (AhRR) and P450 cytochrome (Cyp1A1) in the lung homogenate (Figure 1A). Furthermore, an increased number of AhR^+^ cells in the lung tissue by immunofluorescence (Figure 1B) and by flow cytometry analysis (Figure 1C, Table S1, Supplemental Figures 1, 2) were observed.

Since ozone activates AhR expression, we performed functional tests on the BAL supernatant and lung homogenate from mice exposed to ozone using the H1L1.1c2 luciferase reporter cell line (16) and found an activation of the reporter cell line in the lung homogenate (Figure 1D). These data therefore demonstrate the presence of an AhR-activating moiety in lung supernatant of ozone-exposed mice.

Endogenous metabolites from the host and commensals are involved in AhR activation (15, 16, 22). To identify potential metabolites in our model, lungs from mice exposed to ozone were homogenized, and the supernatants were analyzed by LC-MS. Several known ligands of AhR were assessed (Table S2). Two peaks were observed, the first one corresponding to tryptophan, suggesting an increase of tryptophan metabolism and metabolites that activate AhR, and a second peak corresponding to the lipoxin A4 (LXA4), an anti-inflammatory molecule (44–46) (Figure 1E).

Therefore, ozone exposure induced AhR ligand production in the lung, and we identified LXA4 as a candidate activating AhR.

### AhR Regulates Lung Inflammation, Injury, and Airway Hyperreactivity Upon Chronic Ozone Exposure

**Figure 2:**
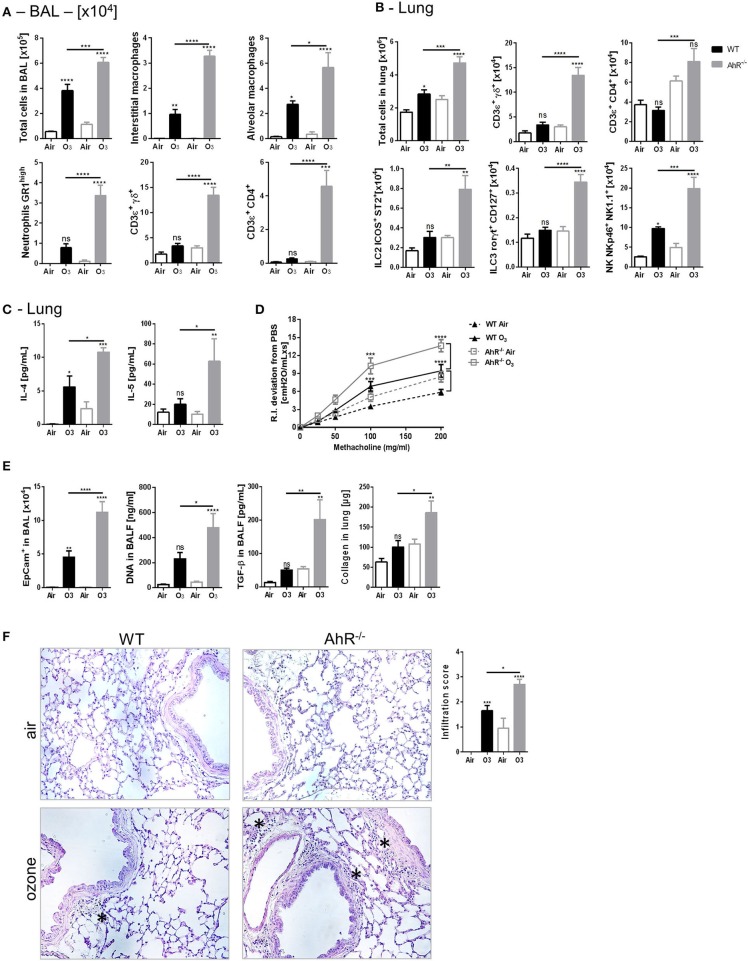
Inflammation, AHR, and remodeling parameters in AhR deficient mice. BAL cells recruitment (total cells, macrophages, neutrophils, and T cells) **(A)**. Lung cells recruitment (total cells, neutrophils, macrophages, ILC2, ILC3, αβ, and γδ T cells) **(B)**, T_H_2 cytokines (IL-4, IL-5) **(C)**, AHR **(D)**, and remodeling parameters (epithelial cells, DNA, and TGF-β in BAL; collagen in lung) **(E)**; lung histology representative pictures (400× magnification, asterisk show cell infiltration) and cell infiltration score **(F)** after ozone exposure in WT and AhR deficient mice. The data are representative for one of two independent experiments with *n* = 5–6 mice per group. Values are expressed as mean ± SEM.

To investigate the role of AhR in ozone induced lung inflammation model, AhR-deficient mice (AhR^−/−^) were exposed to ozone (1.5 ppm for 2 h over 6 weeks). Increased cell infiltration in the lung of AhR^−/−^ mice was observed after ozone exposure in comparison to WT mice (Figures 2A,B). Indeed, in the BAL, an increased number of macrophages, neutrophils, and T cells were detected (Figure 2A) with augmented MPO, AREG, and collagen levels (Supplemental Figure 3A). More particularly, increased absolute numbers of interstitial macrophages, ILC2, ILC3, αβ T cells, γδ T cells, and NK were found in the lung of AhR^−/−^ in comparison to WT mice after ozone exposure (Figure 2B, Supplemental Figure 1). Furthermore, the T_H_2 cytokines and IL-4 and IL-5 levels were higher in the lung from AhR-deficient mice in comparison to WT mice (Figure 2C) with an increased recruitment of IL-5^+^ ILC2 and/or IL-13^+^ ILC2 (Supplemental Figure 3B).

Considering the known effect of ozone on airway hyperresponsiveness, we investigated whether chronic ozone exposure exacerbates airway hyperresponsiveness. Compared to air-exposed mice, ozone-exposed WT mice exhibit a significantly increased lung resistance (RI) in response to methacholine (Figure 2D), which is significantly increased in AhR^−/−^ mice (Figure 2D). Since ozone disrupts the epithelial barrier (41, 47–49), we analyzed the desquamation of epithelial cells by microscopic assessment of the lung integrity. DNA release was used as a marker of cell death in the alveolar space, and TGF-β (50) and collagen deposition were used as indicators of repair with fibrosis in the lung. An increased number of epithelial cells, DNA, and TGF-β levels in BALF were observed (Figure 2E). Collagen was also augmented in the lung homogenate of WT mice, which was further increased in AhR^−/−^ mice (Figure 2E). The microscopic investigations indicated a chronic inflammatory cell infiltration, which was enhanced in the lung of AhR^−/−^ mice in comparison to WT mice (Figure 2F). Mucus-producing cells were increased after ozone exposure, but not different in absence of AhR (data not shown).

Taken together, these data show an increased cell recruitment, airway hyperreactivity, epithelial cell injury, inflammation, and fibrosis in AhR-deficient mice, suggesting that AhR regulates lung inflammation and epithelial damage after ozone exposure.

### AhR Expressed by T Cells Is Critical to Control Ozone Induced Lung Inflammation

The effect of AhR on T cell differentiation and plasticity of T_H_17 and Treg cells has been reported (23). To understand whether AhR solely expressed by T cells is sufficient to regulate ozone induced lung inflammation, AhR-deficient mice in T lymphocytes (AhR^CD4cre^) were generated by crossing AhR^flox/flox^ with CD4^cre^ mice (WT^CD4cre^), and T cell-specific AhR depletion was checked in these mice (Supplemental Figure 4).

**Figure 3:**
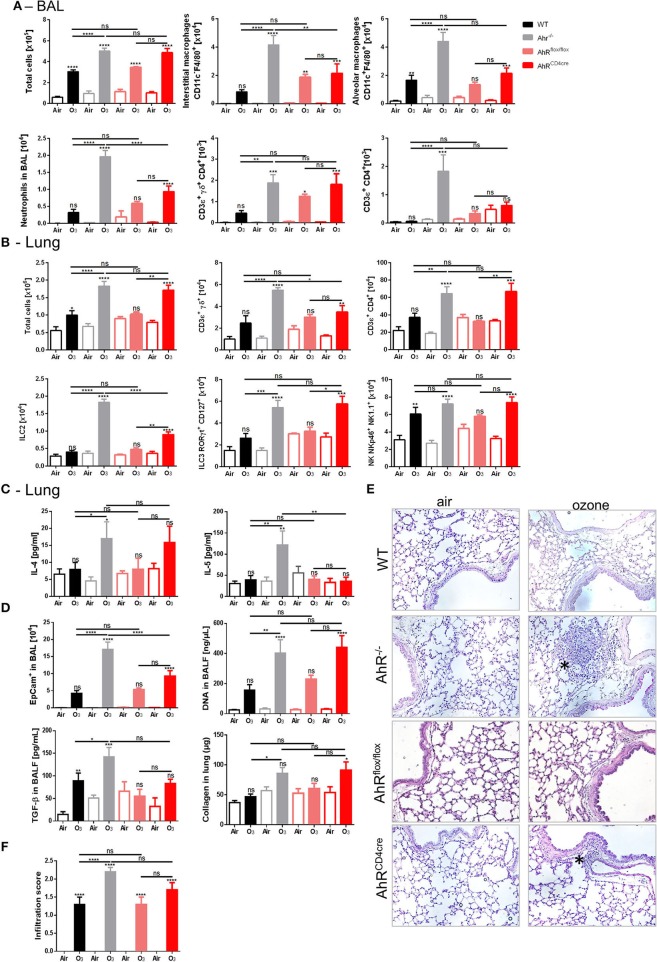
Specific AhR depletion in T cell affects inflammatory parameters. BAL cells recruitment (total cells, macrophages, neutrophils, and T cells) **(A)**. Lung cells recruitment (total cells, neutrophils, macrophages, ILC2, ILC3, αβ, and γδ T cells) **(B)**, T_H_2 cytokines (IL-4 and IL-5) **(C)** and remodeling parameters (epithelial cells, DNA, and TGF-β in BAL; collagen in lung) **(D)**; lung histology representative pictures (400× magnification, asterisks show cell infiltration) **(E)**, and cell infiltration score **(F)** after ozone exposure in WT and AhR deficient mice. The data are representative for one of two independent experiments with *n* = 5–6 mice per group. Values are expressed as mean ± SEM.

Increases of ILC2, ILC3, and CD4^+^ T cells were observed in the lung (Figure 3B) to same extent as in both AhR^CD4cre^ and the AhR^−/−^ mice, in comparison to the AhR^flox/flox^ control mice and WT mice, after ozone exposure, but not for BAL (Figure 3A). In contrast, interstitial macrophages and neutrophils were lower in the BAL in AhR CD4cre mice in comparison to AhR^−/−^ mice, as well as ILC2 and γδ T cells in the lung (Figures 3A,B). Moreover with regards to T_H_2 cytokine level, IL-4 from AhR^CD4cre^ mice was similar to WT mice, but not for IL-5 (Figure 3C).

In addition, the increased epithelial barrier damage observed in the AhR^CD4cre^ mice was similar to AhR^−/−^ mice, as shown by increased DNA release in the BAL and the collagen deposition in the lung (Figure 3D). The epithelial cell desquamation level in the BAL was reduced in the AhR^CD4cre^ mice compared to the AhR^−/−^ mice (Figure 3D). Microscopic observation revealed that inflammatory cell infiltration was comparable in AhR^−/−^ and AhR^CD4cre^ mice (Figures 3E,F). To exclude any dysfunction of AhR by introducing loxP sites in the control AhR^flox/flox^ mice, AhR^flox/flox^ mice were compared with WT mice (Figures 3A–F) and no relevant differences were observed between them, suggesting no impact from genetic modification.

Therefore, the data obtained from AhR^CD4cre^ mice suggest that AhR deficiency in T cells recapitulates partially the data from AhR^−/−^ mice (notably in lung cells recruitment), and hence, AhR expressed in T cells is partially necessary to control ozone-induced inflammation in lung.

### AhR Activation During Chronic Ozone Exposure Suppresses the Recruitment of IL-17A and IL-22 Producing Cells

AhR has a broad-spectrum of biological activity including on the production of IL-17A and IL-22 (22, 51–53). IL-17A is involved in the inflammatory process after acute (28, 29) and chronic ozone exposure (31). IL-22, in view of its protective role in the epithelial barrier, might be involved in ozone injury. Therefore, we assessed the role of AhR on IL-17A and IL-22 production during chronic ozone exposure.

**Figure 4:**
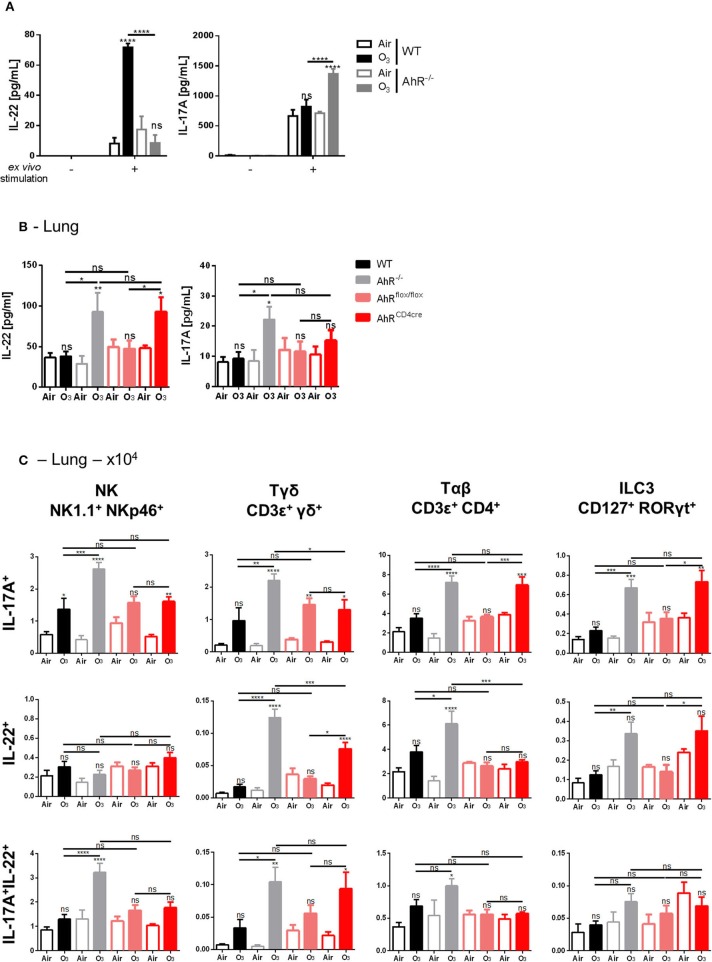
Influence of AhR on IL-17A and IL-22 production. IL-22 and IL-17A release after *ex vivo* stimulation of lung cells **(A)**. Levels of IL-17A and IL-22 in WT and AhR deficient mice **(B)**. Absolute number **(C)** of NK (NK1.1^+^NKp46^+^), ILC3 (CD45^+^Lin^−^CD127^+^Rorγt^+^), αβ T cells (CD45^+^CD3 ε^+^CD4^+^), γδ T cells (CD3ε^+^panγδ^+^), IL-17A^+^, IL-22^+^, and IL-17A^+^IL-22^+^ producing cells. The data are representative for one of independent experiments with *n* = 5–6 mice per group. Values are expressed as mean ± SEM. Statistical significance was defined at a *p*-value **** < 0.0001, *** < 0.001, ** < 0.01, and * < 0.05.

First, the capacity of cells deficient for AhR to produce IL-17A and IL-22 was assessed. With *ex vivo* stimulation of isolated lung cells from WT or AhR^−/−^ mice, enhanced IL-22 production was observed in WT mice, which is abrogated in AhR^−/−^ mice, while the production IL-17A was increased (Figure 4A). These findings suggest that IL-22 and IL-17A may be differentially regulated by AhR. Moreover, increased levels of IL-17A and IL-22 were detected in the lung homogenate of the AhR^−/−^ exposed mice in comparison to ozone exposed WT mice (Figure 4B). After 6 weeks of ozone exposure, IL-17A and IL-22 production was higher in the lung when AhR gene was missing, suggesting an impact of AhR on the recruitment of IL-22-producing cells. This increase was lost for IL-17A in AhR^flox/flox^ and AhR^CD4cre^ mice, but conserved in AhR^CD4cre^ mice for IL-22 (Figure 4B).

To understand the mechanism involved in IL-22 and IL-17 increase, we identified the cell types expressing IL-17A and IL-22 in mononuclear cells isolated from the lungs of ozone-exposed mice. NK, ILC3, αβ, and γδ T cells expressed IL-17A or IL-22 or both IL-17A/IL-22 upon ozone exposure (Figure 4C). IL-17A expression was increased in NK cells, ILC3, αβ, and γδ T cells in AhR^−/−^ mice, in comparison to WT mice, and an increased IL-22 expression was observed in T cells and ILC3 (Figure 4C). In addition, a lower expression of IL-22 in T cells and a slight decrease of IL-17A expression in NK and γδ T cells in AhR^CD4cre^ mice were observed in comparison to AhR^−/−^ mice (Figure 4C). For double positive cells for IL-17A and IL-22, we observed an increase of NK, γδ, and αβ T cells in the AhR^−/−^ mice in comparison to WT mice. A similar phenotype to the one observed in the AhR^−/−^ mice was found for AhR^CD4cre^ mice for the γδ T cells. Therefore, the presence of AhR in CD4^+^ cells is necessary for the recruitment of the IL-17A^+^ αβ T cells and the IL-22^+^ and IL-17A^+^ ILC3 and partially for the IL-22^+^ and of IL-17A^+^/IL-22^+^ γδ T cells (Figure 4C).

Thus, the data suggest that AhR downregulates indirectly IL-22 production via IL-22-producing cells recruitment, like NK, ILC3, and T cells, after chronic ozone exposure. For IL-17A, AhR acts directly on cell production and indirectly on cell recruitment as for IL-22.

### Role of IL-17A and IL-22 Axis Upon Ozone Exposure and AhR Dependence

**Figure 5:**
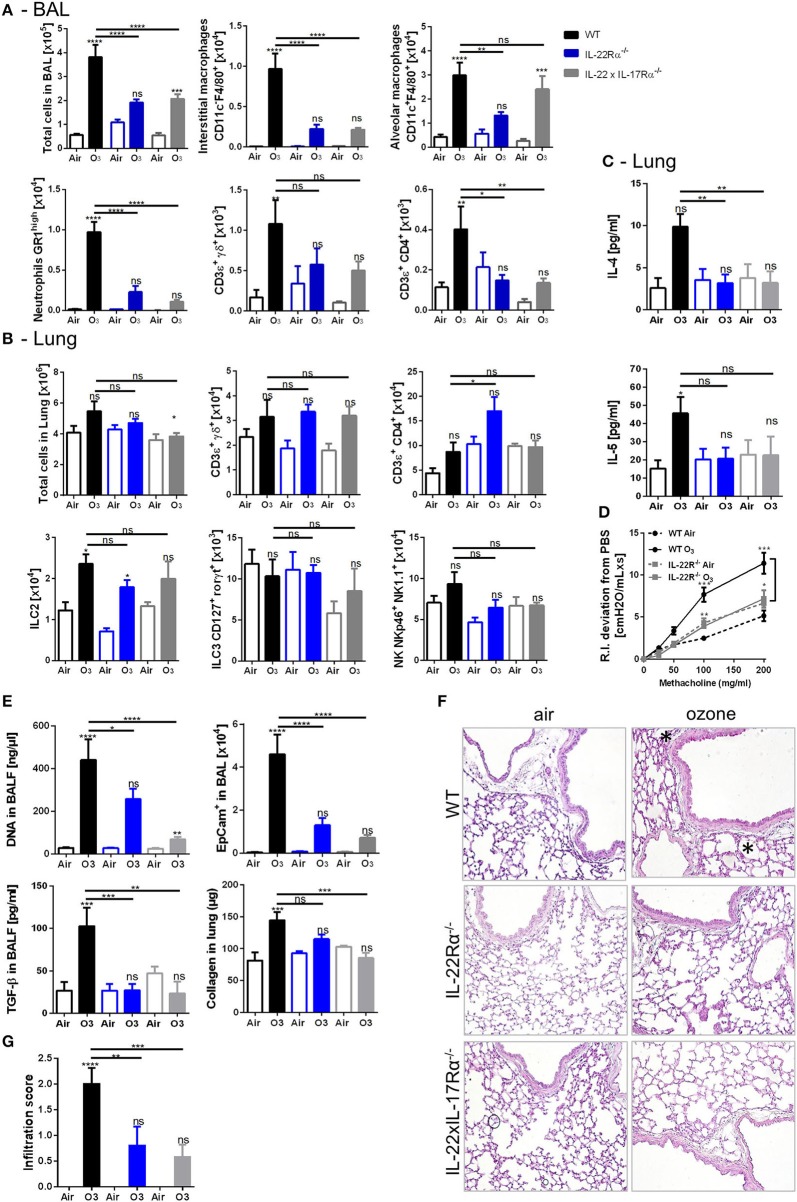
Inflammatory parameters in IL-22R and IL-22/17Rα deficient mice. BAL cells recruitment (total cells, macrophages, neutrophils, and T cells) **(A)**. Lung cell recruitment (total cells, neutrophils, macrophages, ILC2, ILC3, αβ, and γδ T cells) **(B)**, T_H_2 cytokines (IL-4, IL-5) **(C)**, AHR **(D)**, and remodeling parameters (epithelial cells, DNA and TGF-β in BAL; collagen in lung) **(E)**; lung histology (400× magnification, asterisk show cell infiltration) **(F)** and cell infiltration score **(G)** after ozone exposure in WT and AhR-deficient mice. The data are representative of one from two independent experiments with *n* = 5–6 mice per group. Values are expressed as mean ± SEM.

**Figure 6:**
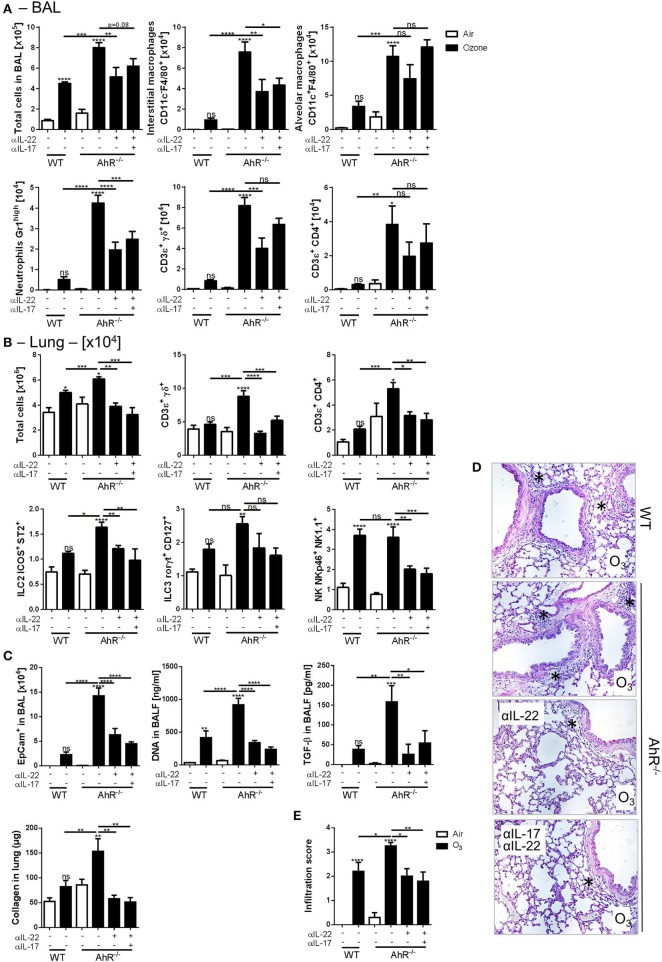
IL-22 and IL-17A neutralization in AhR deficient mice. BAL cells recruitment (total cells, macrophages, neutrophils, and T cells) **(A)**. Lung cell recruitment (total cells, neutrophils, macrophages, ILC2, ILC3, αβ, and γδ T cells) **(B)**, and remodeling parameters (epithelial cells, DNA, and collagen in lung) **(C)**; lung histology (400× magnification; asterisks show cell infiltration) **(D)** and cell infiltration score **(E)** after ozone exposure in WT and AhR-deficient mice. The data are representative of one from two independent experiments with n = 5–6 mice per group. Values are expressed as mean ± SEM.

The possible involvement of IL-17A and IL-22 in dampening the lung inflammation in AhR^−/−^ mice was then investigated. IL-22^−/−^ (Supplemental Figures 6A–F), IL-22Rα^−/−^, and IL-22 × IL-17Rα^−/−^ mice (Figure 5, Supplemental Figure 5) were exposed to ozone, and different lung inflammation parameters were evaluated. Reduced cell infiltration in all gene deficient mice used was observed after chronic exposure (Figures 5A,B, Supplemental Figures 6A,B), with diminished macrophages and neutrophils recruitment in the BAL (Figure 5A), and NK and ILC2 cells in the lung, but not significantly (Figure 5B). Moreover, T_H_2 cytokines, including IL-4 and IL-5, were reduced after chronic ozone exposure in IL-22Rα^−/−^ and in IL-22 × IL-17Rα^−/−^ mice in comparison to WT mice (Figure 5C). Airway hyperresponsiveness was reduced in the IL-22Rα^−/−^ and IL-22^−/−^ mice in comparison to WT mice (Figure 5D, Supplemental Figure 6D). Epithelial cells desquamation in the BAL, DNA, and TGF-β levels in BALF, as well as collagen in the lung were reduced in IL-22Rα^−/−^ and IL-22 × IL-17Rα^−/−^ mice (Figure 5E). Histological analysis of the lung tissue revealed reduced cell infiltration in parenchyma, in the IL-22^−/−^, IL-22Rα^−/−^, and IL-22 × IL-17Rα^−/−^ mice after chronic ozone exposure, in comparison to WT mice (Figures 5F,G). Anti-IL-22 alone is sufficient to recapitulate the phenotype, the anti-IL-17A does not increase the effect observed with the anti-IL-22 (Supplemental Figure 7A). A decrease in interstitial macrophages and γδ T cells recruitment was observed in the BAL (Supplemental Figure 7A), as well as decreased T and NK cells in the lung (Supplemental Figure 7B). Lung remodeling parameters, such as epithelial cells desquamation, DNA release, and collagen production, were reduced in comparison to WT mice (Supplemental Figures 7C,D). Moreover, using the same neutralizing antibodies in AhR-deficient mice, we showed that the increased inflammatory parameters were dependent on IL-22/IL-17A signaling. In the BAL, IL-22 antibody neutralization was sufficient to reduce interstitial macrophages, neutrophils, and T cells, in comparison to what was observed in the AhR deficient mice (Figure 6A). Similar reductions of the T cells, ILC2, ILC3, and NK were observed in the lung (Figure 6B). Moreover, desquamation, DNA release, collagen production, and inflammatory score were reduced in AhR-deficient mice that were treated by anti-IL22 and anti-IL-17A antibodies weekly (Figures 6C–E).

Therefore, the data demonstrated that the absence of IL-22 reduced inflammation, suggesting a pro-inflammatory effect of this cytokine in this model. Since IL-22 expression is enhanced in absence of AhR, we hypothesized that AhR modulate inflammation including the recruitment of IL-22-producing cells, which play a major role in ozone induced lung injury.

## Discussion

Environmental air pollution plays an important role in chronic respiratory diseases (54). Ozone is a major air pollutant contributing to the development of allergic and non-allergic asthma and chronic lung diseases, such as COPD and emphysema.

Here, we report that tryptophan produced upon ozone exposure leads to AhR activation and IL-22 and IL-17A production in the lung, probably through the production of tryptophan metabolites. AhR plays a protective role on lung inflammation, airway hyperresponsiveness, and tissue remodeling after chronic ozone exposure (1.5 ppm, twice a week for 2 h). The protection via AhR had previously been described in several models of lung inflammation including the COPD model induced by cigarette smoke (55, 56) and idiopathic pneumonia syndrome (1) or in the DSS-induced colitis model (57). AhR acts on several pathways, including cell cycle, cytokine production (51, 58), metabolism (20), and the maintenance of ILC3 (19). The type of immune response induced by AhR activation depends on the nature of the ligand. Several studies showed differential cytokine production due to AhR activation after PBMC stimulation with TCDD or PGE2 (22) supporting this hypothesis.

After ozone exposure, we identified two molecules released in the lung: tryptophan and LXA4, which potentially activate AhR. AhR plays a key role of tryptophan metabolism. Since the 1980s, several studies reported a role of tryptophan metabolites in the lung, including in cancer (59) and chronic inflammatory lung diseases affecting the parenchyma, such as sarcoidosis and idiopathic fibrosis (1, 60). The indole/tryptamine pathway could induce the production of several indole from tryptophan that may activate AhR. For example, in the intestine, IAld is known to induce the activation of AhR and the production of IL-22 (53). In such conditions, IAld is produced by microbiota, in particular the *Lactobacilli*. Lung/gut microbiota in the context of ozone exposure had been recently studied (61); moreover, ozone is commonly used in sterilization protocols, and it has an impact on plants (62), suggesting an impact of ozone on microbiota. Stress of the lung microbiome (63) induced by inflammatory cells and epithelial cells desquamation after ozone exposure may lead to an increased release of tryptophan in the lung that can be converted in various metabolites, but we cannot exclude an initial synthesis in gut. Moreover, we identified another AhR ligand, the lipoxin A4. The amount of lipoxin A4 was five times higher than the tryptophan itself in the lung tissue after chronic ozone exposure. This class of ligand has been described for the first time in 1999 (64). Lipoxin 44 could be produced in different cell types, as neutrophils, eosinophils, alveolar macrophages, and epithelial cells (65). Lipoxin A4 effects are mediated through its binding to FPR2/ALX membrane receptor or to AhR. Lipoxins are known to participate to inflammation resolution. In experimental asthma, lipoxins regulate NK or ILC2 activation (66), and in experimental psoriasis model induced by imiquimod, they reduce IL-17A and IL-22 production via HMGB1 modulation (67).

Here, we show that, AhR activation induced the recruitment of several immune cells, including macrophages, γδ T cells, αβ T cells, NK, ILC2, and ILC3 (Graphical Abstract). Upon chronic ozone exposure in AhR-deficient mice, we observed a type 2 response, as demonstrated by the production of T_H_2 cytokines (IL-4 and IL-5). This response is associated with an increased IL-6 production. Airway hyperresponsiveness after chronic ozone exposure, a key feature of ozone exposure (28), is significantly enhanced in absence of AhR, as expected in a TH2 response. In the intestine, AhR maintenance is involved in the recruitment (19) of ILC3; however, the link between AhR and the ILC3 have not been investigated in the lung yet. Compared to the ILC2, ILC3 cells are very few in the lungs. But after ozone exposure, the quantity of ILC2 and ILC3 are very similar. Thus, we hypothesize that ILC3 had migrated from intestine to other tissues, particularly in the lung tissue after chronic ozone exposure. The deletion of AhR, in the CD4^+^ cells, controls the recruitment of the ILC3 and CD4^+^ T cells and is partly involved in the recruitment of the ILC2. Various direct or indirect mediators can drive this effect on different cell populations. Moreover, AhR may act on iNKT function (68), on the generation of Treg (69) or T_H_17 cells (23), and in the balance between T_H_1 and T_H_2 (70) as AhR activation suppresses the T_H_2 differentiation.

Furthermore, AhR regulates IL-17A and IL-22 production (Graphical Abstract). Here, in AhR-deficient mice, IL-17A, and IL-22 levels were increased as well as the amount of NK, ILC3, and γδ T cells expressing IL-17A and IL-22. By using AhR^CD4cre^ mice we observed that the AhR-specific deficiency in T cells influenced ILC3 numbers and increased their capability to produce IL-17A and IL-22. IL-17A is known to be a pro-inflammatory cytokine that can collaborate with IL-22 to induce inflammation (71, 72). We hypothesized that the protective effect of AhR is related to its capacity to repress the recruitment of the cells secreting IL-17A and IL-22 likely mediated the production of an endogenous ligand induced by ozone. This repression could be driven by the LXA4. Indeed, AhR deficient mice present a reduced LXA4 expression (73). As shown by Zhang et al. (31), IL-17A is pro-inflammatory in the context of chronic exposure to ozone, confirmed in our lab with utilization of IL-17Rα^−/−^ mice (data not shown). In the IL-22Rα^−/−^ mice, we observed a reduction of cell infiltration and T_H_2 cytokines production. Moreover, airway hyperresponsiveness and tissue remodeling parameters (epithelial cells, DNA release, and collagen deposition) were reduced. Thus, our data confirm the pro-inflammatory role of both IL-17A and IL-22 in this model, but our data suggest that the phenotype observed in AhR^−/−^ mice is only dependent of IL-22.

In conclusion, ozone exposure induces tryptophan metabolites and LXA4 that activates AhR. This activation likely control cell recruitment, T_H_2 and T_H_17/22 response, airway hyperresponsiveness and tissue remodeling. Altogether, our data show that AhR plays a protective role upon chronic exposure to ozone by modulating IL-22-producing cells recruitment leading to the control inflammation (Graphical Abstract).

## Data Availability Statement

All datasets generated for this study are included in the article/Supplementary Material.

## Ethics Statement

The animal study was reviewed and approved by The French Institutional Committee under agreement CLE CCO 2015-1088.

## Author Contributions

CM, FB, LF, IM, CC, and MS conducted the experiments. CM, BR, and DT designed the experiments. CM, BR, DT, CC, JS, VQ, HS, AC-M, and LD wrote the paper or had a critical regard on manuscript.

### Conflict of Interest

DT and LF are employees at ArtImmune. The remaining authors declare that the research was conducted in the absence of any commercial or financial relationships that could be construed as a potential conflict of interest.

## Abbreviations

AhR: Aryl hydrocarbon receptor
AhRR: AhR repressor
AREG: Amphiregulin
ARNT: Aryl hydrocarbon nuclear translocator
BAL: Broncho alveolar lavage
IDO: Indoleamine 2,3-Dioxygenase
ILC: Innate lymphoid cells
iNKT: invariant NK T cells
MPO: Myeloperoxidase
NK: Natural killer
PGE2: Prostaglandin E2
PBMC: Peripheral blood mononuclear cells
WT: Wild type (C57BL/6).
