# Supplementary material for: Ozone-Induced Aryl Hydrocarbon Receptor Activation Controls Lung Inflammation via Interleukin-22 Modulation
Source: Front Immunol. 2020 Feb 25;11:144. doi: 10.3389/fimmu.2020.00144 (PMC7053361; doi:10.3389/fimmu.2020.00144)
Supplement: Supplementary file 1 [file Data_Sheet_1.pdf]

Supplementary Material

Table 1: Antibodies used for the FACS staining

Antibodies	Fluorochrome	Supplier	Ref	Concentration (µg/mL)
CD11b	PerCP-Cy5.5	BD	550993	2
CD11c	APC-Cy7	BD	561 241	2
CD127	PE-CY7	BD	560733	2
NKp46	V450	BD	560764	2
CD3e	PerCp Cy5.5	BD	551163	2
CD3e	APC-Cy7	BD	557596	2
CD4	V500	BD	560782	2
CD4	PE-Cy7	BD	552775	2
CD45	PE	BD	553081	2
CD45	V450	BD	560501	2
CD45	V500	BD	561487	10
CD45R/B220	PerCP-Cy5.5	BD	552771	2
EpCam	APC	BD	563478	2
EpCam	PE	BD	563477	2
F4/80	V450	eBioscience	48-4801-82	2
FcεRIα	PerCP-Cy5.5	eBiosciences	134320	2
GR1/Ly6G/6C	PE-Cy7	BD	552985	0,2
ICOS	PE-Cy7	eBiosciences	25-9942-80	2
NK-1.1	APC-CY7	BD	560618	2
pan gd T Cell	FITC	BioLegend	118106	5
Siglec-F	PerCP-Cy5.5	BD	565526	2
ST2	FITC	mdbioproducts	101001F	10
IL-22	PE	eBiosciences	12-7221-82	10
AhR	APC	R&D systems	IC6697A	
IL-13	APC	eBiosciences	50-7133-82	10
IL-17A	FITC	BD	560220	10
IL-17A	PE	BD	559502	10
IL-22	APC	eBiosciences	17-7222-82	10
IL-5	PE	BD	554395	10
RORγt	APC	eBioscience	17-6981-82	10
RORγt	Percp-Cy5.5	BD	562683	10
ROS	FITC	Life Technologies	D399	5 µmol/L

Table 2: AhR ligands detected

AhR Ligand	Formula	MW (g/mol)	ref
Bilirubin	$C_{33}H_{36}N_4O_6$	585	(Stejskalova et al. 2011)
Biliverdin	$C_{33}H_{34}N_4O_6$	583,25	(Stejskalova et al. 2011)
Cinnabarinic acid	$C_{14}H_8N_2O_6$	301	(Lowe et al. 2014)
dFICZ	$C_{21}H_{13}O_2N$	312	(Machowinski et al. 2006)
DIM	$C_{17}H_{14}N_2$	241	(Bjeldanes et al. 1991)
Equilenin	$C_{18}H_{18}O_2$	267	(Stejskalova et al. 2011)
FICZ	$C_{19}H_{12}N_2O$	285	(Machowinski et al. 2006)
Hemin	$C_{34}H_{32}ClFeN_4O_4$	561	(Stejskalova et al. 2011)
ICZ	$C_{18}H_{10}N_2$	255	(Bjeldanes et al. 1991)
Indigo	$C_{16}H_{10}N_2O_2$	263	(Stejskalova et al. 2011)
Indirubin	$C_{16}H_{10}N_2O_2$	263	(Stejskalova et al. 2011)
Indole-3-acetaldehyde	$C_{10}H_9NO$	160	(Bjeldanes et al. 1991)
Indole-3-acetic acid	$C_{10}H_9NO_2$	176	(Miller 1997)
Indole-3-acetonitrile	$C_{10}H_8N_2$	157	(Bjeldanes et al. 1991)
Indole-3-aldehyde	C_9H_7NO	146	(Zelante et al. 2013)
Indoxyl-3-Sulfate	$C_8H_7NO_4S$	214	(Schroeder et al. 2010)
ITE	$C_{14}H_{10}N_2O_3S$	287	(Song et al. 2002)
Kynurenic acid	$C_{10}H_7NO_3$	190	(Stejskalova et al. 2011)
Kynurenine	$C_{10}H_{12}O_3N_2$	209	(Mezrich et al. 2010)
PG2	$C_{20}H_{32}O_6$	369	(Stejskalova et al. 2011)
Lipoxin A4	$C_{20}H_{32}O_5$	353	(Stejskalova et al. 2011)
Phenazine	$C_{12}H_8N_2$	181	(Zelante et al. 2014)
Phtiocol	$C_{11}H_8O_3$	189	(Zelante et al. 2013)
Skatole	C_9H_9N	132	(Weems and Yost 2010)
TCDD	$C_{12}H_{14}Cl_4O_2$	322	(Poland and Knutson 1982)
Trp	$C_{11}H_{12}N_2O_2$	205	(Zelante et al. 2013)
Tryptamine	$C_{10}H_{12}N_2$	161	(Miller 1997)
Xanthurenic acid	$C_{10}H_7NO_4$	206	(DiNatale et al. 2010)

Supp Figure 1: Gating strategy used for the FACS analysis

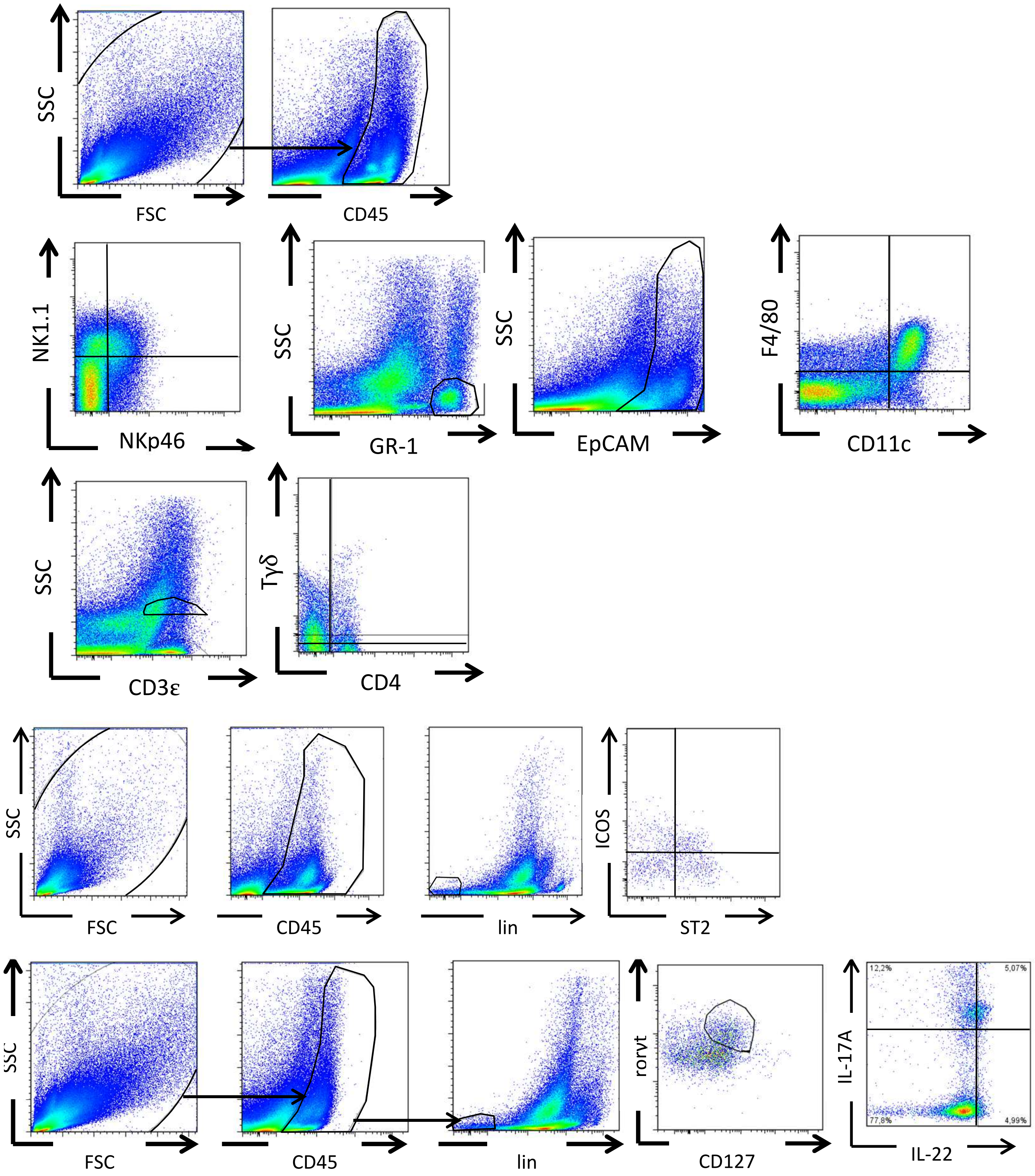

Strategy to FACS analysis for CD45+/- cells, for NK (CD45+NKp46+NK1.1+), neutrophils (CD45+GR-1high), epithelial cells (CD45-EpCAM+), macrophages (F4/80+CD11c+/-), CD4+ T cells (CD45+CD3ε+CD4+), γδ T cells (CD45+CD3e+γδ+), ILC2 (CD45+lin-ICOS+ST2+),and ILC3 (CD45+lin-CD127+Rorγt+).

Supp Figure 2: AhR expression after chronic ozone exposure

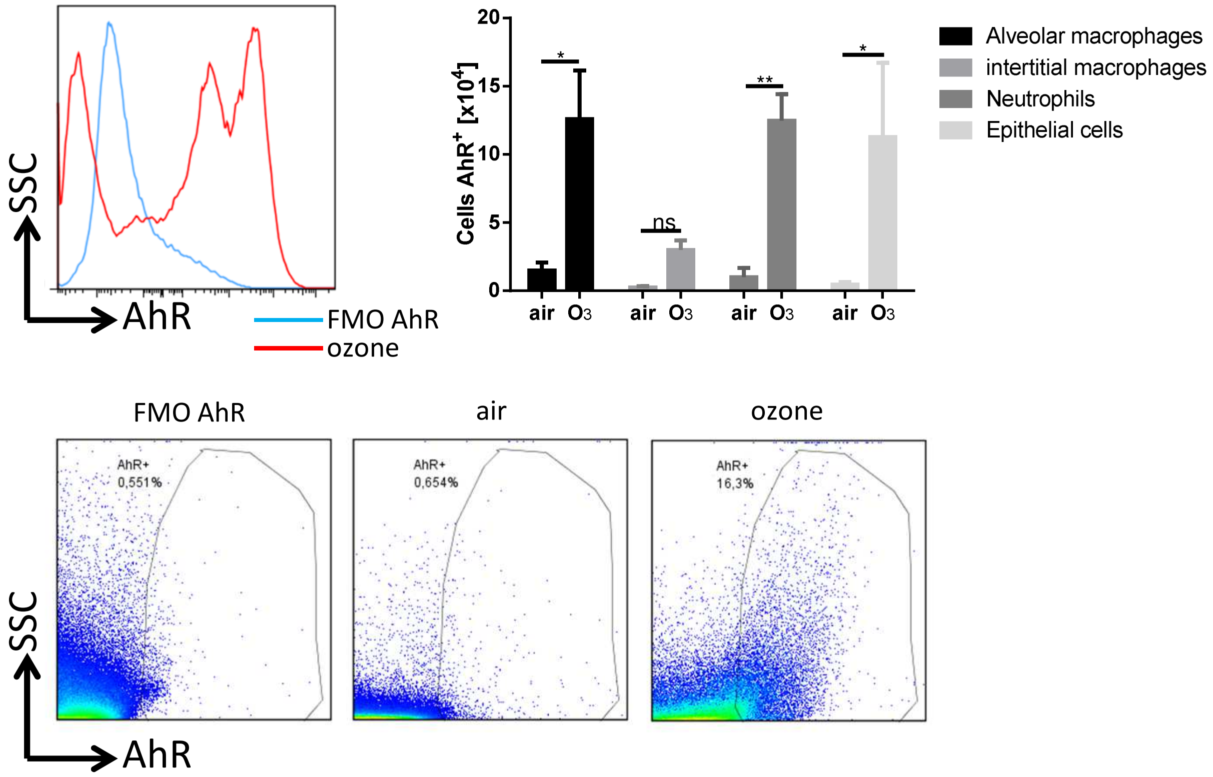

AhR expression on histogram or dotplot in FMO, air, and ozone WT mice. AhR expression in macrophages, neutrophils, and epithelial cells. The data are representative of one of two independent experiments with n = 5 mice per group. Values are expressed as mean ± SEM.

Supplemental Figure 3: AhR deficiency affects BAL inflammatory parameters and lung ILC2 recruitment

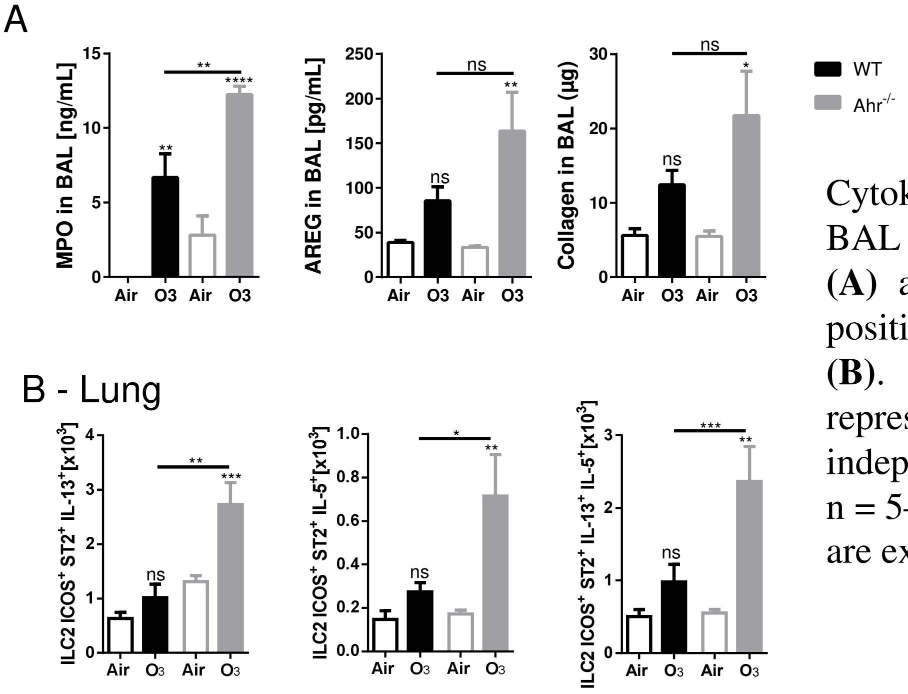

Cytokines/protein levels in the BAL (MPO, AREG, Collagen) **(A)** and recruitment of ILC2 positive for IL-5 and/or IL-13 **(B)**. The data are representative of one of two independent experiments with n = 5–6 mice per group. Values are expressed as mean ± SEM.

Supplemental Figure 4: Validation of AhR depletion in CD4+ cells in AhRCD4cre mice

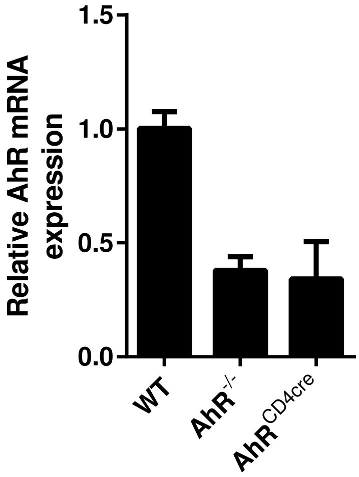

AhR expression in splenocytes of WT, AhR-/- and AhRCD4cre mice was detected by qPCR. Experiments represent two mice per group. Values are expressed as mean ± SEM.

Supplemental Figure 5: IL-22Rα and IL-22xIL-17Rα deficiency affects BAL inflammatory parameters

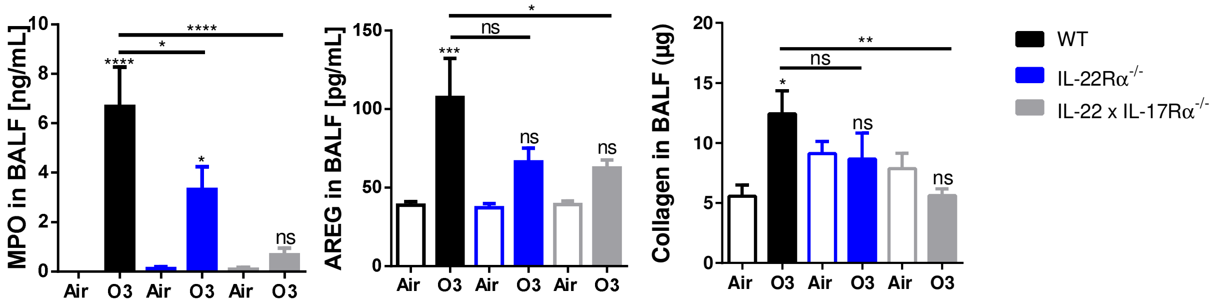

Cytokines/protein levels (MPO, AREG, and Collagen) in the BAL. Data are pooled from two experiments, with 5–6 mice per group. Values are expressed as mean ± SEM.

Supplemental Figure 6: Influence of IL-22 deficiency on inflammatory parameters

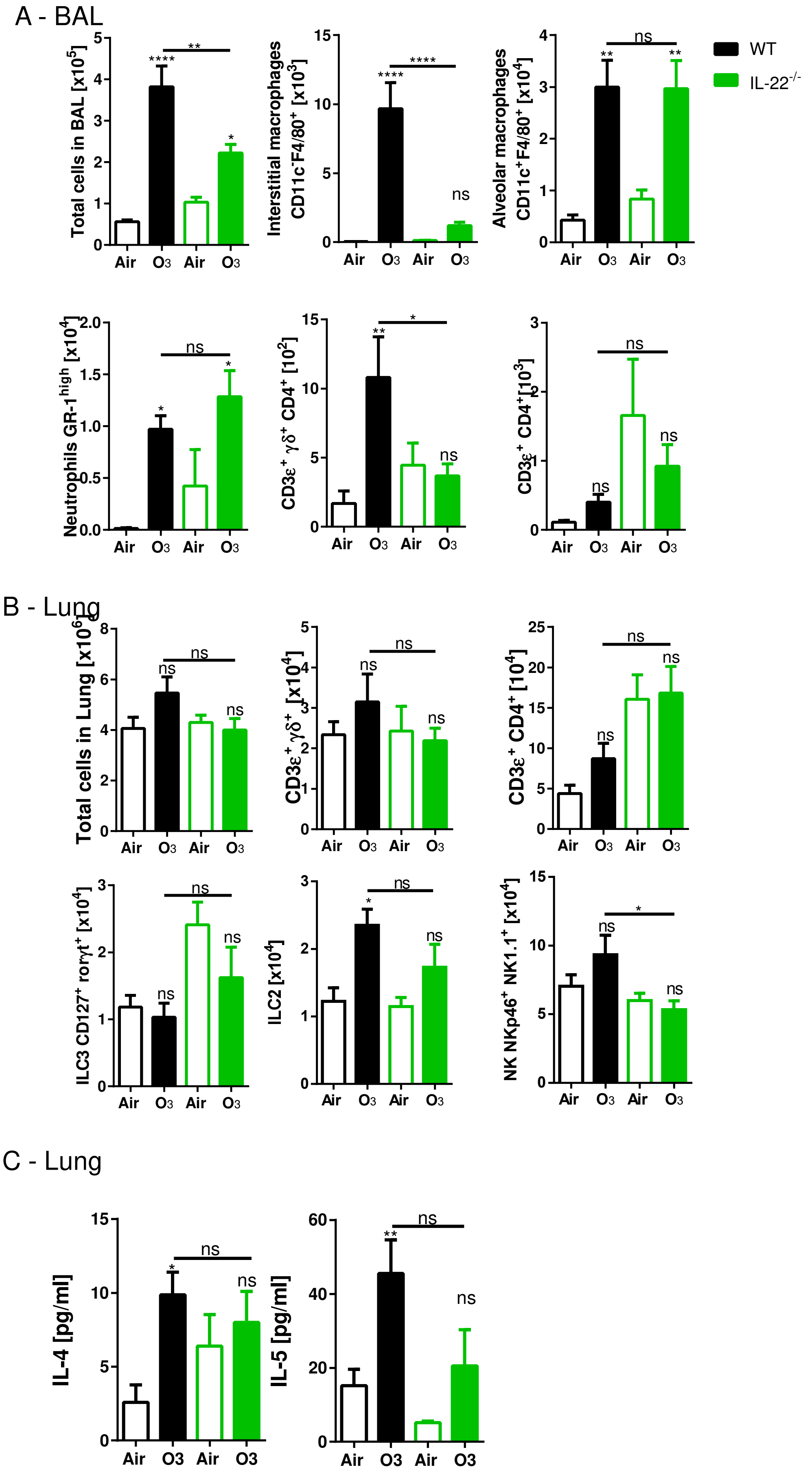

D

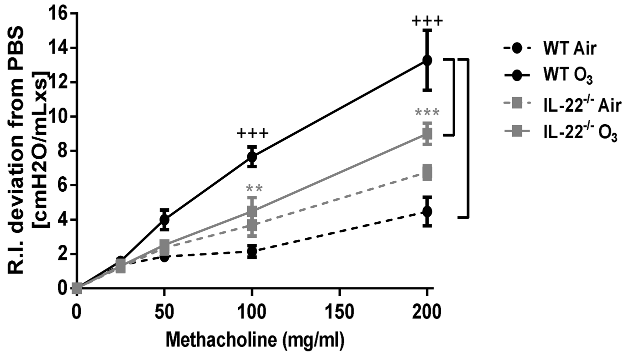

E

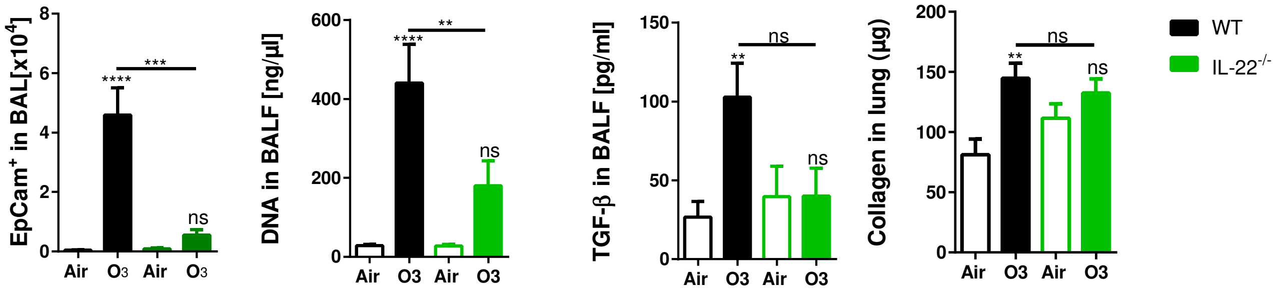

F

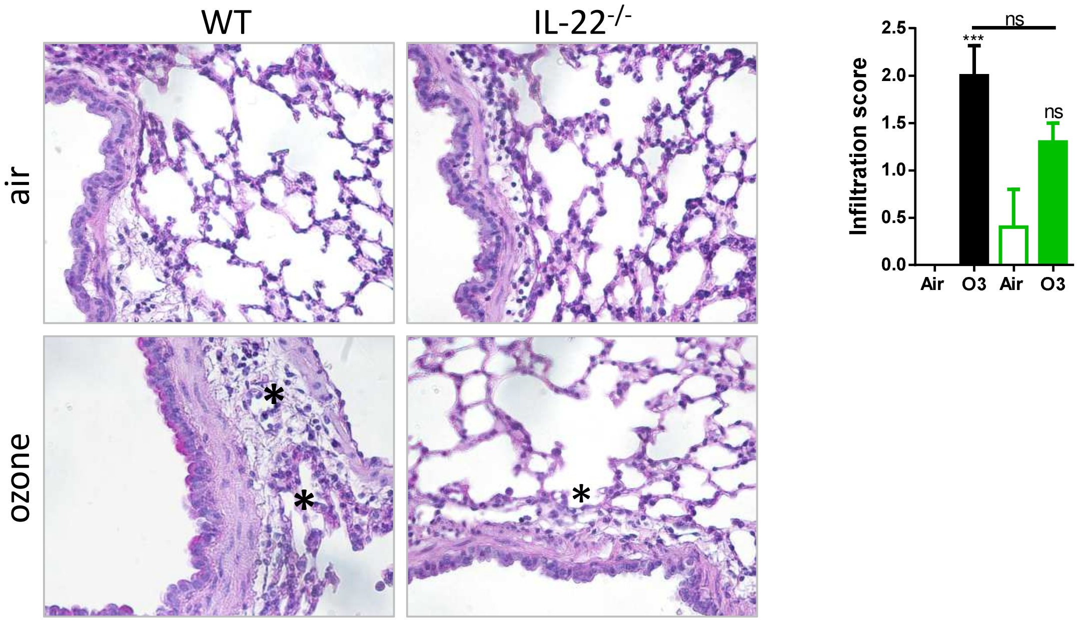

BAL cells recruitment (total cells, macrophages, neutrophils and T cells) **(A)**. Lung cell recruitment (total cells, neutrophils, macrophages, ILC2, ILC3, αβ, and γδ T cells) **(B)**, TH2 cytokines (IL-4, IL-5) **(C)**, AH **(D)**, and remodeling parameters (epithelial cells, DNA and TGFβ in BAL; collagen in lung) **(E)**; lung histology and cell infiltration score (400× magnification, asterisk show cell infiltration) **(F)** after ozone exposure in WT and IL-22-deficient mice. Experiments are pooled of two experiments and repeated twice, with n = 5–6 mice per group. Values are expressed as mean ± SEM.

Supplementary Figure 7: Influence of IL-22 neutralization in WT mice

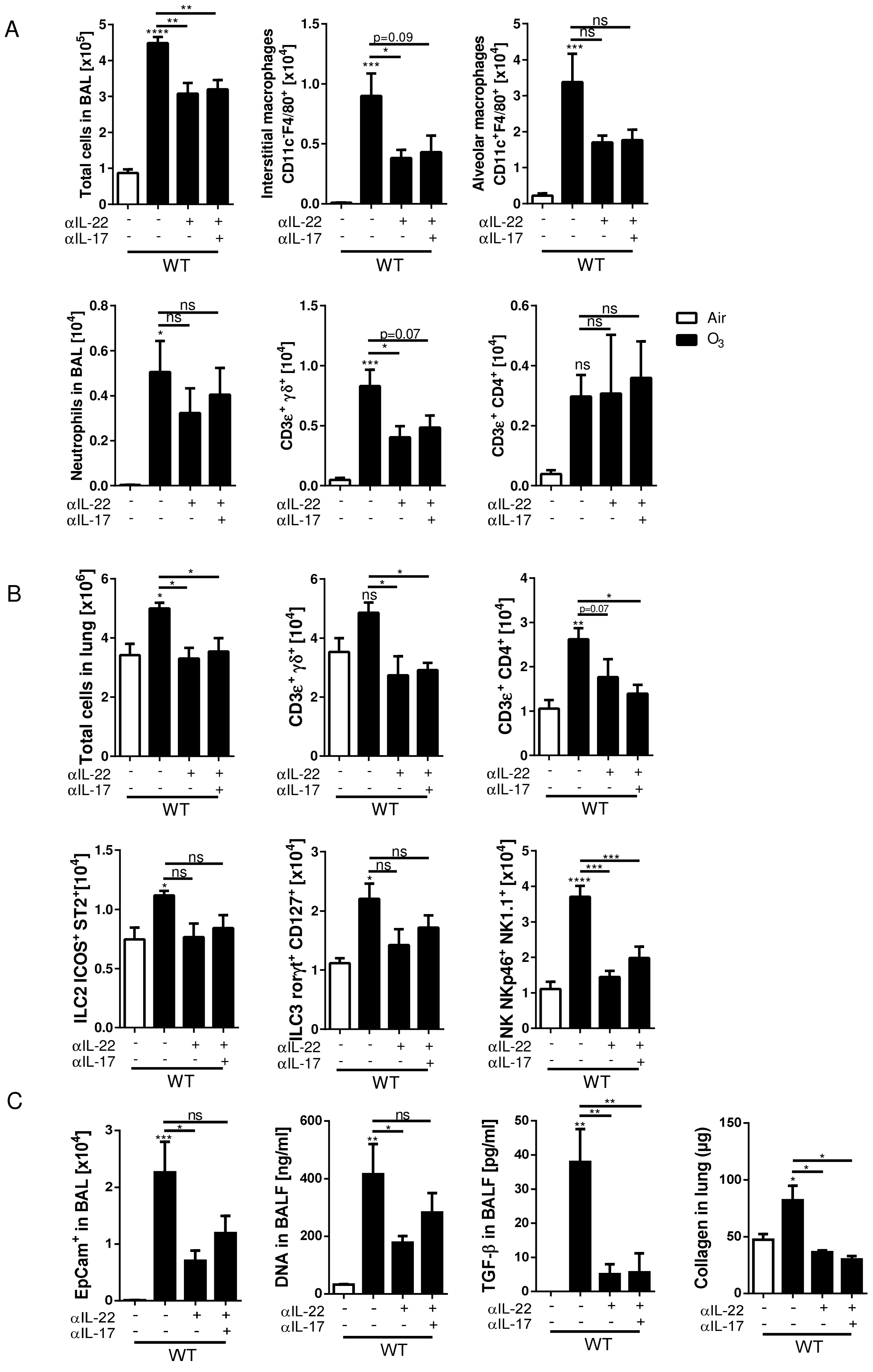

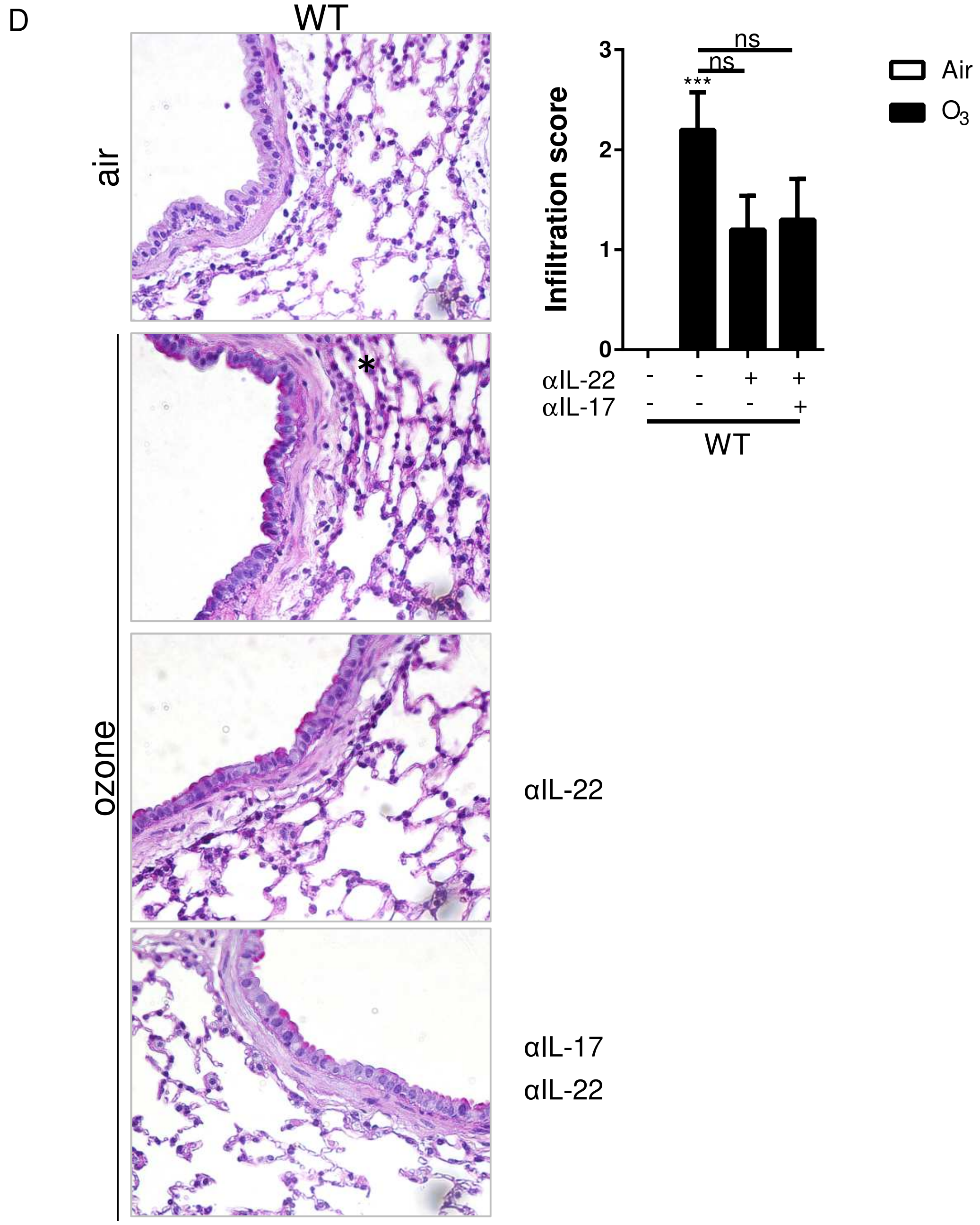

BAL cells recruitment (total cells, macrophages, neutrophils, and T cells) **(A)**. Lung cells recruitment (total cells, neutrophils, macrophages, ILC2, ILC3, αβ, and γδ T cells) **(B)** and remodeling parameters (epithelial cells, DNA, and collagen in lung) **(C)**; lung histology (400× magnification, asterisks show cell infiltration), and cell infiltration score **(D)** after ozone exposure in WT, with or without αIL-22 and αIL-17A antibodies administration. The data are representative of one from two independent experiments with n = 5–6 mice per group. Values are expressed as mean ± SEM.
